# Water-Soluble Pristine C_60_ Fullerenes Inhibit Liver Fibrotic Alteration and Prevent Liver Cirrhosis in Rats

**DOI:** 10.1155/2020/8061246

**Published:** 2020-02-13

**Authors:** Halyna Kuznietsova, Natalia Dziubenko, Vasyl Hurmach, Iryna Chereschuk, Olexandr Motuziuk, Olexandr Ogloblya, Yuriy Prylutskyy

**Affiliations:** ^1^Taras Shevchenko National University of Kyiv, Volodymyrska str., 64, 01601 Kyiv, Ukraine; ^2^Lesya Ukrainka Eastern European National University, Volya Avenue, 13, 43025 Lutsk, Ukraine

## Abstract

Liver cirrhosis is an outcome of a wide range of liver chronic diseases. It is attributed to oxidative stress; therefore, antioxidant usage could be a promising treatment of that. So, exploring the impact of effective free radical scavenger pristine C_60_ fullerenes on liver fibrosis and cirrhosis and their ability to interact with main growth factor receptors involved in liver fibrogenesis was aimed to be discovered. We used N-diethylnitrosamine/carbon tetrachloride-induced simulations of rat liver fibrosis (10 weeks) and cirrhosis (15 weeks). Pristine C_60_ fullerene aqueous colloid solution (C_60_FAS) was injected daily at a dose of 0.25 mg/kg throughout the experiment. Liver morphology and functional and redox states were assessed. C_60_ fullerenes' ability to interact with epidermal, vasoendothelial, platelet-derived, and fibroblast growth factor receptors (EGFR, VEGFR, PDGFR, and FGFR, respectively) was estimated by computational modeling. We observed that C_60_FAS reduced the severity of fibrosis in fibrotic rats (0.75 vs. 3.0 points according to Ishak score), attenuated the hepatocyte injury, normalized elevated blood serum alkaline phosphatase (ALP) and lactate dehydrogenase (LDH), and mitigated oxidative stress manifestation in liver tissue restoring its redox balance. When applied to cirrhotic animals, C_60_FAS reduced connective tissue deposition as well (2.4 vs. 5.4 points according to Ishak score), diminished ALP and LDH (by 16% and 61%), and normalized conjugated and nonconjugated bilirubin, restoring the liver function. Altered liver lipid and protein peroxides and glutathione peroxidase activity were also leveled. Within a computer simulation, it was shown that C_60_ fullerenes can block hinge prohibiting ATP binding for EGFR and FGFR and thus blocking associated signal pathways. This ability in addition to their antioxidant properties may contribute to C_60_ fullerene's antifibrotic action. Thus, C_60_FAS may have a substantial therapeutic potential as an inhibitor of liver fibrosis and cirrhosis.

## 1. Introduction

Liver cirrhosis is the end-stage condition of a wide variety of chronic liver diseases and an increasing cause of morbidity and mortality worldwide. Its 1-year mortality ranges from 1% to 57% depending on the stage. To date, the only treatment of developed cirrhosis is liver transplantation [1].

Liver fibrosis, as a pathological process, is characterized by the growth of connective tissue without changing the gland structure wherein the liver lobules are not altered, but there are wide bands of fibrous connective tissue around them. The cirrhosis develops during fibrosis progress: the liver lobes become replaced with connective tissue with the formation of cirrhotic nodes. This leads to a significant decrease in the liver functional activity and the development of liver failure. Furthermore, liver fibrosis and cirrhosis are considered as main risk factors of nonviral etiology of hepatocellular carcinoma (HCC) development. Thus, HCC arises from chronic liver inflammation, fibrosis, and eventually cirrhosis in 70-80% of cases [2].

Liver fibrosis is an “excessive” healing with the formation of an excess amount of connective tissue incorporated into the liver parenchyma. This process is accompanied by extracellular matrix overproduction and/or its incomplete degradation [3]. The liver chronic injury is the trigger of fibrogenesis. Usually, it is accompanied by excessive production of reactive oxygen species (ROS), lipid peroxidation products, and proinflammatory cytokines, which cause activation of hepatic stellate cells (HSCs) that proliferate with the formation of myofibroblasts. Hence, oxidative stress can play a key role in HSC activation, fibrogenesis initiation, progression, and transition to cirrhosis [4]. HSCs after being activated require growth factors for their proliferation, as any cells do. Indeed, growth factor receptor signaling is essential for HSC proliferation and subsequent liver fibrogenesis and attracts the attention as promising target of antifibrotic treatment [5, 6].

The main etiological factors of liver fibrosis and cirrhosis are as follows: alcohol, storage diseases, hepatitis viruses, and hepatotoxic drugs. There is no specific remedy for liver fibrosis to date. Some compounds having therapeutic activity against liver fibrosis are undergoing preclinical and I-II phases of clinical trials. They include (1) the monoclonal antibodies and low molecular inhibitors of key signaling pathways involved in the regulation of inflammation, HSC life cycle, and collagen metabolism [7] (however, these substances are highly specific, i.e., target the only link of a separate signaling pathway) and (2) the broad-spectrum agents exhibiting antioxidant, anti-inflammatory, hepatoprotective, and antilipotoxic activities such as ursolic, ursodeoxycholic, and 24-norursodeoxycholic acids, resveratrol, and silymarin [4]. However, these agents are rather supplements, the positive effect of which is observed only in combination with other therapeutics.

The biocompatible nanoparticles attract the attention of researchers as correcting tools of a wide range of diseases including liver fibrosis [8]. They include, in particular, metal-based nanoparticles (Au, Ag, and Ce) and their oxides, liposomes, and micelles, which being conjugated with antifibrotic agents (cisplatin, doxorubicin, and curcumin), are capable of delivering them into the liver. Carbon-based nanostructure—C_60_ fullerene—is one of the most powerful free radical scavengers [9, 10]: 1 molecule of С_60_ is able to bind up to 34 radicals depending on their size [11]. No toxic effect of water-soluble pristine C_60_ fullerenes in the dose range of 75–150 mg/kg was observed on a mouse model (the LD_50_ value was 721 mg/kg) [12]. Water-soluble C_60_ fullerene is able to accumulate in rat liver [13] and prevent its acute toxic injury [14]. Additionally, in our previous studies, we demonstrated its ability to prevent not only acute but also chronic liver injury [15], to serve as an anti-inflammatory agent [16], and to inhibit liver fibrosis on acute and chronic cholangitis models [17] applying in small doses. Moreover, water-soluble C_60_ fullerene is capable of reducing collagen deposition in the lungs of bleomycin-induced pulmonary fibrosis mice [18]. However, these effects were observed after a relatively short period of C_60_ fullerene action due to the “rapid” development of the simulated pathologies (from 1-2 days till 4 weeks). Effects of this nanocompound on liver pathology, which requires much longer time to develop (several months), have not been investigated yet. Therefore, the aim of this work was to explore the impact of water-soluble pristine C_60_ fullerene on liver fibrosis and cirrhosis in the diethylnitrosamine-carbon tetrachloride rat model, as well as its ability to interact with main growth factor receptors involved in liver fibrogenesis using *in silico* study.

## 2. Materials and Methods

### 2.1. Preparation and Characterization of Pristine C_60_ Fullerene Aqueous Colloid Solution

The highly stable pristine C_60_ fullerene aqueous colloid solution (C_60_FAS; purity > 99.5%, concentration 0.15 mg/ml) was prepared according to the method of [19, 20]. Briefly, it is based on the transferring of C_60_ fullerenes from toluene to an aqueous phase with the help of ultrasonic treatment.

The atomic force microscopy (AFM) was performed to determine the size of C_60_ fullerene particles (their aggregates) in the aqueous solution. Measurements were done with the “Solver Pro M” system (NT-MDT Spectrum Instruments, Russia). A drop of investigated solution was transferred on the atomic-smooth substrate to deposit layers. Measurements were carried out after complete evaporation of the solvent. A freshly broken surface of mica (V-1 grade, SPI supplies, West Chester, PA, USA) was used as a substrate. Measurements were carried out in a semicontact (tapping) mode with AFM probes of the RTPESPA150 (Bruker, 6 N/m, 150 kHz) type.

We used the dynamic light scattering (DLS) and zeta potential measurements for ascertaining the hydrodynamic size and electrokinetic potential of the prepared C_60_FAS. The tested volume of C_60_FAS was 1.5 ml. Measurements were conducted on a Zetasizer Nano ZS90 (Malvern, Malvern, Worcestershire, UK) at 25°C. Results were evaluated using the Smoluchowski approximation, which is known to be rigorously valid only for spherical-like particles.

### 2.2. Animals

Studies were conducted using 48 Wistar male rats with the initial body weight of 120 ± 10 g, which were kept in a vivarium of Taras Shevchenko National University of Kyiv. After randomization, 8 animals were housed per plastic cage on softwood chip bedding, maintained under constant conditions (12 h light/dark cycle, 50% humidity at 20-22°C), and fed a standard diet and tap water *ad libitum*. All experiments were conducted in compliance with bioethics principles, legislative norms, and provisions of the European Convention for the Protection of Vertebrate Animals Used for Experimental and Other Scientific Purposes (Strasbourg, 1986), General Ethical Principles for Experiments on Animals, adopted by the First National Bioethics Congress (Kyiv, 2001), and approved by an institutional review committee.

### 2.3. Design of the Study

**Figure 1:**
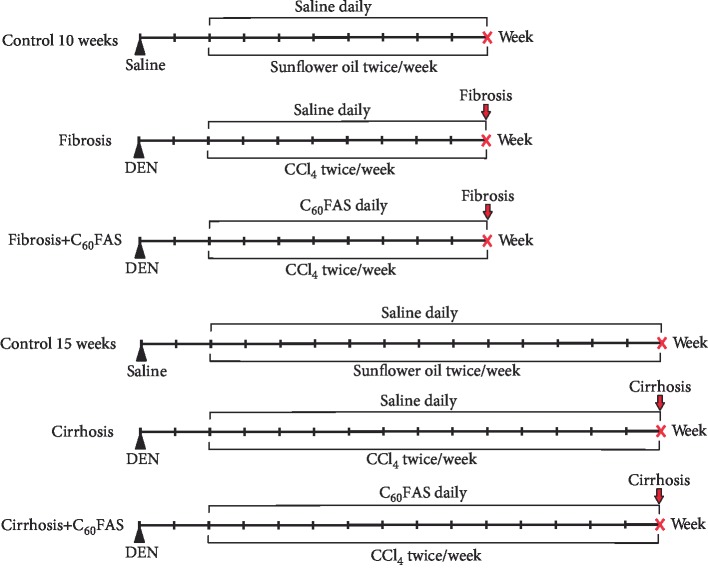
The scheme of the experiment. Experimental groups were as follows (from top to bottom): control 10 weeks (*n* = 8); fibrosis 10 weeks, fibrosis and cirrhosis development (*n* = 8); fibrosis+C_60_FAS 10 weeks, C_60_FAS was applied for 8 weeks (*n* = 8); control 15 weeks (*n* = 8); cirrhosis 15 weeks, definitive cirrhosis, initiation of cell malignant degeneration (*n* = 8); and cirrhosis+C_60_FAS 15 weeks, C_60_FAS was applied for 13 weeks (*n* = 8). Doses and solvents: DEN: 200 mg/kg in saline (total volume 0.1 ml), CCl_4_: 1 ml/kg in sunflower oil (total volume 0.2-0.6 ml depending on body weight), C_60_FAS: 0.25 mg/kg (0.2-0.5 ml depending on body weight), saline: 0.1 ml (instead of DEN) or 0.2-0.5 ml (instead of C_60_FAS), sunflower oil: 0.2-0.6 ml depending on body weight.

Fibrosis and cirrhosis were simulated as follows: an inducer of fibrogenesis and malignant degeneration N-diethylnitrosamine (DEN, 200 mg/kg) was injected intraperitoneally in saline. After 2 weeks, carbon tetrachloride (CCl_4_) applications were started to stimulate the regeneration process. CCl_4_ which served as hepatotoxin was injected subcutaneously twice a week in the amount of 1 ml/kg body weight dissolved in sunflower oil in the ratio of 1 : 1 till the end of the study. Changes in the liver correspond to hepatic inflammation (2-6 weeks from the start of the study, i.e., the introduction of DEN), advanced fibrosis and cirrhosis (8-12 weeks from the start of the study), and malignant degeneration of liver cells (14-16 weeks from the start of the study) [21, 22]. C_60_FAS was administered daily intraperitoneally in a volume corresponding to the dose of C_60_ fullerene 0.25 mg/kg, which is considered as safe and effective as evidenced by our previous studies [15–17], starting 2 weeks after DEN administration (i.e., simultaneously with hepatotoxin CCl_4_) for 8 and 13 weeks to explore its effect on the development of fibrosis and cirrhosis, respectively. Comparison groups were induced by appropriate solvents instead (Figure 1).

In 24 h after the last dose, the animals were sacrificed by inhalation of CO_2_ and subsequent cervical dislocation.

### 2.4. Liver Assays

#### 2.4.1. Macroscopic Assay

After the sacrifice, the liver was assessed using the following scoring [23]: 0: reddish brown in color, soft in consistency, smooth surface; 1: the liver was enlarged, friable, soft in consistency; 2: hepatic hyperemia; 3: an enlarged liver with white focal areas of necrosis; 4: an enlarged liver with white multifocal areas of necrosis; 5: hepatic steatosis, the liver was soft, yellow, greasy, and enlarged; 6: the liver is mottled red with bile stained areas, of normal or increased size; 7: contains visible nodules and fibrosis; 8: micronodular, yellow, fatty, enlarged; 9: macronodular, brown, nongreasy, shrunken, cirrhosis; 10: an explanted liver showing small single granule bodies; 11: an explanted liver showing large single granule bodies; 12: an explanted liver showing many small granular bodies; 13: an explanted liver showing many large granular bodies.

#### 2.4.2. Histological Assay

Liver samples were harvested immediately after the sacrifice, fixed in Bouin mixture for 7 days, embedded in paraffin, cut into 5 *μ*m thick sections, stained with hematoxylin and eosin (H&E) according to standard methods [24], and examined under the light microscope. The severity of liver disease in rats was evaluated histologically on H&E-stained sections in a blinded fashion, in which pathologists were unaware of the treatment group. The general state of the tissue, the typical/atypical histological structure, the shape and structure of cells, staining, intensity of inflammatory process (stroma and gland tissue infiltration by leucocytes), vasculature, and the severity of fibrosis were analyzed. Severity of fibrosis was assessed according to Ishak scoring [5]: 0: no fibrosis; 1: fibrous expansion of some portal areas, with or without short fibrous septa; 2: fibrous expansion of most portal areas, with or without short fibrous septa; 3: fibrous expansion of most portal areas, with occasional portal to portal bridging; 4: fibrous expansion of most portal areas, with marked portal to portal bridging as well as portal areas to central bridging; 5: marked bridging with occasional nodules; 6: possible to establish cirrhosis.

### 2.5. Biochemical Assays

#### 2.5.1. Blood Assays

The blood for biochemical analysis was collected immediately after the sacrifice from the femoral vein, left for 20 min to form a clot, and then centrifuged 8 min at 1500 g. Blood serum was collected and used immediately for determination of alanine aminotransferase (ALT), aspartate aminotransferase (AST), alkaline phosphatase (ALP), lactate dehydrogenase (LDH), total (conjugated+nonconjugated) and direct (conjugated) bilirubin (as liver injury and functional state markers), urea, creatinine (as kidney functional state markers), and *α*-amylase (as pancreas functional state marker). We used standard reagent kits (Diagnosticum Zrt, Hungary).

#### 2.5.2. Liver Tissue Assays

The liver was washed with saline followed by phosphate-buffered saline (PBS) containing 1 mM ethylenediaminetetraacetic acid (EDTA) and 0.4 mM phenylmethylsulphonyl fluoride (PMSF, serine proteases inhibitor) having pH 7.0 and rapidly frozen at -70°C. After being thawed, samples were gently homogenized in PBS containing 1 mM EDTA and 0.4 mM PMSF, filtered through four layers of cheesecloth, centrifuged at 10000 g for 15 min to pelletize the nuclei and mitochondria. Supernatants were collected and used for analysis. Total protein was estimated using standard reagent kit (Diagnosticum Zrt, Hungary). Malonic dialdehyde (MDA), protein carbonyl groups (PCG) and reduced glutathione (GSH) levels, intracellular superoxide dismutase (SOD), catalase (CAT), glutathione peroxidase (GP), and total glutathione-S-transferase (GST) activities as markers of the liver redox status were measured spectrophotometrically and expressed per mg protein.

Lipid peroxidation was estimated by reaction with thiobarbituric acid as described by [25]. Absorbance of chromogen was determined at 532 nm; the extent of lipid peroxidation was expressed as MDA using a molar extinction coefficient of 1.56 × 10^5^ M^–1^ · cm^–1^.

Protein oxidation was estimated by reaction of oxidized amino acid residues with 2,4-dinitrophenylhydrazine according to [26]. The stable product 2,4-dinitrophenylhydrazone was quantified spectrophotometrically at 370 nm; the extent of oxidized proteins (PCG) was expressed as 2,4-dinitrophenylhydrazone using a molar extinction coefficient of 2.2 × 10^4^ M^–1^ · cm^–1^.

Level of GSH was assessed based on its ability to be oxidized by 5,5′-dithiobis-2-nitrobenzoic acid resulting in the formation of glutathione disulfide and 5-thio-2-nitrobenzoic acid, having the maximum of absorbance at 412 nm [27]. GSH level was quantified using calibration curve.

SOD activity was assessed by its ability to inhibit the reduction of nitro blue tetrazolium (NBT) by riboflavin as described by [28]. Reaction of O_2_^−^ generation (and NBT recovery to formazan) was initiated by bright sunshine for 10 min, and then, the absorbance was measured at 540 nm. One unit of SOD activity was defined as the amount of enzyme required to cause 1% inhibition of NBT reduction.

CAT activity was assessed by Н_2_О_2_+molybdenum salt method according to [29]. Reaction was initiated by adding of 0.03% Н_2_О_2_ solution to the sample and stopped after 10 min by adding of 4% ammonium molybdate. Absorbance of chromogen was determined at 410 nm. CAT activity was expressed as velocity of Н_2_О_2_ fission using a molar extinction coefficient of 22.2 M^–1^ · cm^–1^.

GP activity was assessed by measuring unconsumed GSH after its addition to reaction mixture and incubation with t-butyl hydroperoxide [30]. GSH level was determined as described above.

Total GST activity was assessed by measuring the conjugation of 1-chloro-2,4-dinitrobenzene with GSH, which is accompanied by an increase of absorbance at 340 nm. Molar extinction coefficient of 9600 M^–1^ · cm^–1^ was used [31].

### 2.6. *In Silico* Assay

Epidermal, vasoendothelial, fibroblast, and platelet-derived growth factor (EGF, VEGF, FGF, and PDGF, respectively) signaling plays a pivotal role in HSC activation, proliferation, and liver fibrogenesis. Inhibition of EGF, VEGF, FGF, and PDGF receptors (EGFR, VEGFR, FGFR, and PDGFR, respectively) has been shown to attenuate hepatic fibrosis [6]. Therefore, we aimed to explore the ability of C_60_ fullerene to interact with these growth factor receptors.

#### 2.6.1. Homology Modeling

We used kinase domains of EGFR (Protein Data Bank (PDB) ID: 5X2K [32]), FGFR (PDB ID: 4XCU [33]), PDGFR (PDB ID: 5GRN [34]), and VEGFR (PDB ID: 4AG8 [35]). Considering that all of the above structures have amino acid gaps, homology modeling was used to obtain better results for both molecular docking and molecular dynamics (MD). Using the UniProtKB online service, 100% similar amino acid sequences were selected for each structure (P00533 for 5X2K (EGFR), P22455 for 4XCU (FGFR), P16234 for 5GRN (PDGFR), and P35968 for 4AG8 (VEGFR)). Further, all missing elements were completed using the SWISS MODEL server [36].

#### 2.6.2. Molecular Docking

The C_60_ molecule and above kinase domain structures were used for modeling. Molecular docking assay was performed using flexible ligand and rigid protein. We applied the algorithm of systematical docking (SDOCK+), built in the QXP (quick explore) package (this method demonstrates all possible conformations of the studied structures with the minimal value of root mean square deviation) [37]. We generated 300 potentially possible complexes of C_60_ fullerene with every protein. The 10 best of those were selected for the next stage, using a scoring function, built in the QXP package [38]. The optimal structures of generated complexes were selected using the following criteria: 1: the area of contacting surfaces of protein and ligand; 2: the distance between protein and ligand; and 3: the binding energy characteristics for the complex.

#### 2.6.3. MD Simulation

To assess the stability/conformational changes of obtained complexes, we conducted the short MD (500 ps). All calculations were performed using Gromacs 5.1.3 software [39] and Charmm36 force field [40]. The resulting complexes were protonated according to Gromacs 5.1.3-implemented “ingh” function. The C_60_ fullerene topology was obtained using a SwissParam server [41]. The system was initially placed in the center of the square box and filled with water molecules (TIP3P). For free rotation of the complex, the distance between that and the walls of the box was 9 Å. To simulate the cellular environment, an appropriate amount of Nа^+^/Cl^−^ ions was added (their concentration in the box was set as 0.15 M). Then, the energy was minimized, followed by two equilibration steps: (1) NVT (constant number of particles, volume, and temperature) and (2) NPT (constant number of particles, pressure, and temperature). Finally, the MD simulation duration 5 ns was performed.

### 2.7. Statistical Analysis

Homogeneity of variance was assessed using the Levene test. Statistical analysis of the data was performed using one-way analysis of variance (ANOVA) with the Tukey post hoc test. The difference was considered statistically significant at *p* < 0.05.

## 3. Results

### 3.1. C_60_FAS Characterization

The monitoring of the C_60_ fullerene size distribution in aqueous solution is important for controlling the degree of aggregation which may influence its bioactivity and toxicity [42–46]. The prepared C_60_FAS was characterized by AFM and DLS techniques.

**Figure 2:**
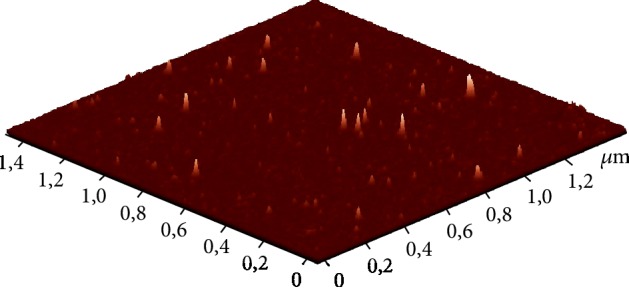
AFM image of the C_60_ fullerene nanoparticles on the mica surface (concentration 0.15 mg/ml).

The AFM study of C_60_ fullerene film deposited from an aqueous solution revealed a high degree of molecule dispersion in solution. It turned out that the used C_60_FAS contains both single C_60_ fullerene (objects with a height of ~0.7 nm in Figure 2) and its labile nanoaggregates with a size of 1.3-70 nm. The majority of C_60_ molecules were located chaotically and separately along the surface or in the form of bulk nanoclusters consisting of several tens of C_60_ molecules [47] (objects with a height of 1.3-2 nm in Figure 2). Thus, the C_60_FAS applied in our experiments is a typical colloidal nanofluid.

The DLS method has shown that C_60_FAS (concentration 0.15 mg/ml) contains nanoparticles of hydrodynamic size from ~1 to 100 nm, a considerable part (42%) of which are nanoparticles with a diameter of 80 nm. In addition, the registered polydispersity index value 0.24 indicates a moderately dispersed distribution of the tested C_60_FAS, which agrees well with the above AFM data.

The stability of the used C_60_FAS was evaluated by the zeta potential measurement. This value was shown to be -23.4 mV. Such a high (by absolute value) zeta potential for the tested C_60_FAS indicates high stability (low tendency for nanoparticle aggregation over time) and its suitability for further biological research.

### 3.2. 10-Week Fibrosis

**Figure 3:**
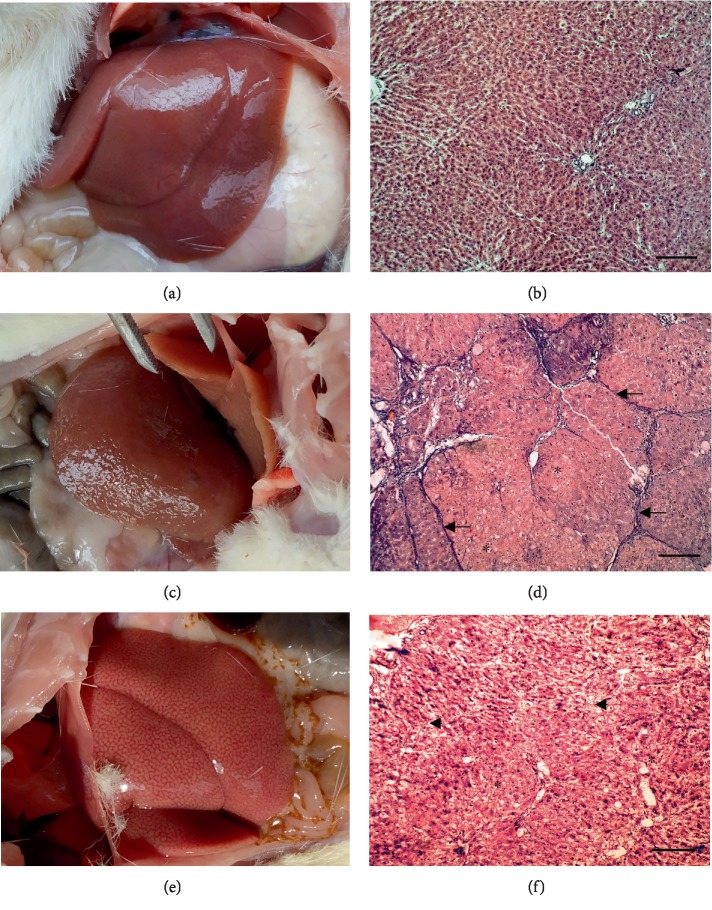
(a, c, e) Representative rat livers at the time of the sacrifice and (b, d, f) H&E staining of liver tissue; magnification ×100, scale 200 *μ*m. (a, b) Control 10 weeks; (c, d) fibrosis 10 weeks; (e, f) fibrosis+C_60_FAS 10 weeks. Arrows: portal-portal linking septa; asterisk: hepatocellular hypertrophy; arrowhead: necrotic areas.

Autopsies of all nontreated 10-week fibrosis animals demonstrated the signs of steatohepatosis and micronodular fibrosis. At the H&E-stained slides, accumulation of connective tissue around the portal tracts and well-developed portal-portal linking septa were observed. Lipid dystrophy, eosinophilic cellular alterations, hepatocellular hypertrophy, and some ground-glass hepatocytes occurred as well. All animals demonstrated liver fibrosis (Figure 3 and Table 1). Although ALT, AST, and conjugated and nonconjugated bilirubin did not alter compared to controls, ALP and LDH activity increased by 1.6 and 3.2 times (*p* < 0.05), respectively. Observed changes (growth of ALP and LDH with unaltered ALT, AST, and bilirubin) may indicate intrahepatic cholestasis, biliary cirrhosis, and even liver cell neoplastic transformation [48, 49]. Blood serum urea also increased by 26% (Table 2).

Liver SOD and CAT activities and GSH level in nontreated animals which experienced 10-week fibrosis were maintained at the control level. However, levels of MDA and PCG tended to go up by 33% (*p* > 0.1) and 280% (*p* = 0.012), respectively, and GP tended to go down by 64% (*p* = 0.048), suggesting the intensification of lipid and protein peroxidation and exhaustion or depression of enzymatic antioxidant defense system. But GST activity also increased by 140% (*p* = 0.049) (Table 2).

C_60_FAS caused the reduction of liver damage score by 12% (*p* = 0.027 compared to nontreated animals), signs of micronodular fibrosis occurred but less pronounced. Histologic examination of liver sections revealed minor fibrosis in half of C_60_FAS-treated animals (4/8) manifested by a slight accumulation of connective tissue around some of the portal tracts without forming septa. Another half of the experimental group (4/8) had no signs of fibrosis at all. However, micro- and macrovesicular fat vacuoles in the parenchyma and areas of necrosis were observed in periportal zones. Fibrosis severity was diminished (by 75%, *p* < 0.001 compared to nontreated animals, difference against control is statistically insignificant) (Figure 3 and Table 1).

Treatment with C_60_FAS restored blood serum ALP and LDH. However, ALT, AST, creatinine, and urea raised up by 4, 3.7, 1.35, and 1.4 times, respectively (*p* < 0.05). This might indicate inhibition of fibrogenesis and neoplastic degeneration but hepatocyte injury and renal failure as well. Nevertheless, liver functional activity corresponded to control, as evidenced by bilirubin levels. C_60_FAS also reversed GST, GP, and PCG in controls and increased GSH by 82% compared to that in nontreated animals (*p* > 0.1). However, SOD and CAT activities tended to go down (Table 2).

### 3.3. 15-Week Cirrhosis

**Figure 4:**
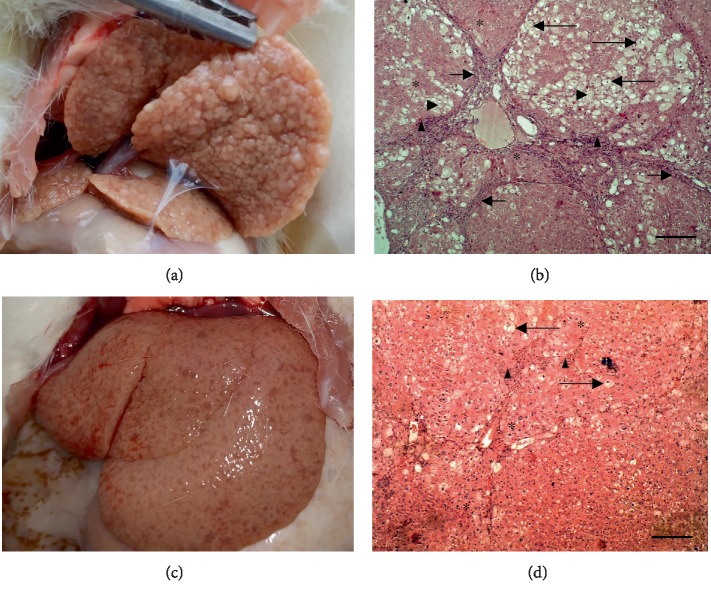
(a, c) Representative rat livers at the time of the sacrifice and (b, d) H&E staining of liver tissue; magnification ×100, scale 200 *μ*m. (a, b) Cirrhosis 15 weeks; (c, d) cirrhosis+C_60_FAS 15 weeks. Arrows: portal-portal linking septa; long arrows: balloon dystrophy; asterisk: hepatocellular hypertrophy; arrowhead: necrotic areas; triangle: inflammatory cell infiltrate.

At the stage of 15 weeks, liver injury and fibrosis development progressed in all animals. Liver autopsies of nontreated animals revealed macronodular fibrosis and cirrhosis; 5 among 8 rats had single tumor nodes (1 per animal). Analyzing H&E slides, we observed well-developed cirrhosis: thick well-developed portal-portal and portal-central fibrous septa surrounding islets of liver parenchyma, hepatocyte lipid and balloon dystrophy with necrotic areas, blood vessel overflow (suggesting portal hypertension), a lot of ground-glass hepatocytes, and hepatocellular hypertrophy occurred. Normal liver tissue was virtually absent (Figure 4 and Table 1).

Blood serum ALP and LDH in these animals dramatically increased (by 4.5 and 28.5 times, respectively, *p* < 0.001); ALT, AST, and conjugated and nonconjugated bilirubin also grew up by 2.9, 2.8, 2.3, and 1.7 times, respectively (*p* < 0.05), suggesting cirrhosis, cell neoplastic degeneration [48–50], hepatocyte destruction, and liver failure (Table 2). Liver GSH diminished (by 69%) and MDA elevated (by 2.2 times) (*p* < 0.05), evidencing probable intensification of lipid peroxidation and depletion of antioxidant defense system meaning oxidative stress. GST activity tended to go up and GP one tended to go down (*p* > 0.1); hereby, changes were similar to those at 10-week fibrosis.

C_60_FAS treatment attenuated liver damage by 15% as evidenced by signs of micronodular fibrosis only. Tumor nodes were observed only in 2 among 8 rats (1 tumor node per animal). Fibrous tissue accumulation around portal tracts occurred, whereas portal-portal linking septa were not observed in most animals. Thus, fibrosis severity was mitigated by 56% (*p* = 0.001 compared to nontreated animals, *p* = 0.004 compared to control). However, similar to C_60_FAS 8-week treatment, lipid and balloon dystrophy and necrotic areas were expressed in portal zones. Hepatocellular hypertrophy and accumulation of inflammatory cells in periportal areas also occurred (Figure 4 and Table 1). C_60_FAS diminished ALP and LDH (by 16% and 61%, respectively, compared to nontreated animals) and normalized conjugated and nonconjugated bilirubin. But ALT and AST remained unchanged compared to those of nontreated animals; creatinine and urea were also elevated (by 24% and 87%, respectively) (Table 2). Obtained data suggest that C_60_FAS suppressed liver fibrogenesis and malignant cell degeneration and normalized liver functional activity. However, hepatocyte injury and renal failure also occurred. Liver MDA was restored to control, as well as GST and GP. GSH level remained lower compared to control; moreover, SOD activity decreased (by 47%) (Table 2).

### 3.4. *In Silico* Study

**Figure 5:**
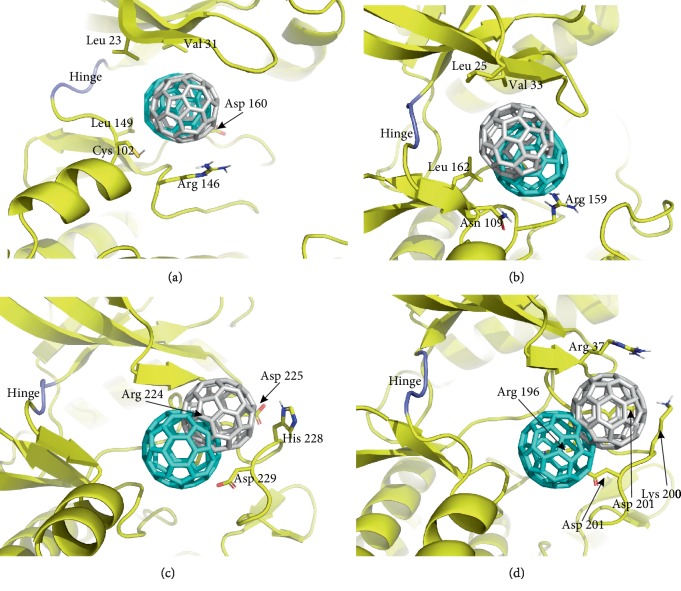
Interaction of C_60_ fullerene with ATP-binding sites of EGFR (a), FGFR (b), PDGFR (c), and VEGFR (d). Protein: yellow; C_60_ fullerene: blue (molecular docking) and grey (MD).

Our main idea was to bring the C_60_ fullerene as close as possible to hinge (Figure 5) to block ATP binding with the corresponding kinase domains. In cases of EGFR and FGFR, C_60_ fullerene was docked close to hinge, which may allow preventing ATP binding. PDGFR and VEGFR failed to dock C_60_ fullerene at a short distance from hinge. Therefore, C_60_ fullerene was docked into the ATP-binding site at a considerable distance from hinge to test its ability to move to hinge during MD (Figure 5).

MD was used to evaluate the stability and possible minimal rearrangements of obtained complexes. According to the obtained results, we found that the mobility for EGFR slightly differed from all other complexes and ranged 0.16-0.41 nm. The mobility range for FGFR was 0.13-0.26 nm and for PDGFR and VEGFR was 0.14-0.27 and 0.12-0.23 nm, respectively. In turn, the mobility of C_60_ fullerene in all cases was almost the same and ranged 0.004-0.008 nm. Despite C_60_ fullerene's low mobility, some displacements of that were observed. A slight C_60_ fullerene shift (0.3 nm) and its gradual displacement from the ATP-binding site were observed for EGFR. However, C_60_ fullerene was firmly wedged between the two *β*-sheets, which make this binding site, and formed cation-*π* and SH-stacking interactions with Arg 146 and Cys 102, respectively (Figure 5). A similar situation was observed in the case of FGFR: C_60_ fullerene slightly shifted (3.8 nm) but did not displace from the ATP-binding site. C_60_ fullerene was also wedged between the two *β*-sheets and formed stable cation-*π* interactions with Arg 159 and Asn 109 (Figure 5). In addition, it should be noted that in both cases C_60_ fullerene formed a significant amount of steric interactions with the amino acid environment of the above proteins.

For PDGFR and VEGFR, a similar interaction with C_60_ fullerene was observed, which, however, differed from EGFR and FGFR. So, C_60_ fullerene cannot interact with surrounding molecular groups in close proximity to hinge. Despite C_60_ fullerene's minor mobility, its significant displacement occurred: on 4.9 nm for PDGFR and on 6.1 nm for VEGFR. C_60_ fullerene was wedged by stacking and cation-*π* interactions between Arg 224 and His 228, respectively, in the case of PDGFR, and by cation-*π* interactions between Arg 196, Arg 37, and Lys 200 in the case of VEGFR (Figure 5).

Obtained data demonstrate the theoretical ability of C_60_ fullerene to block hinge prohibiting ATP binding for EGFR and FGFR. In turn, for PDGFR and VEGFR, the initial theoretical results suggest C_60_ fullerene's inability to block hinge and, consequently, to prevent interaction of kinase domains with ATP. Thus, C_60_ fullerene cannot regulate their operation. Finally, since no significant fluctuations in the total energy of obtained complexes were observed (data not shown), we could conclude that they are stable.

## 4. Discussion

We have shown that C_60_FAS-treated animals which experienced liver fibrosis (10 weeks) and cirrhosis (15 weeks) demonstrated marked reduction of liver damage and connective tissue deposition and partial normalization of liver enzymes. These results indicate inhibition of fibrogenesis and partial restoration of liver function. However, signs of hepatocyte injury and renal failure still occurred. Moreover, there were some evidences of liver malignant degeneration in settings of cirrhosis (LDH strong increase [48]) and its inhibiting by C_60_FAS. HCC develops in chronic inflammatory and cirrhotic environment independent of the primary etiological factor, and exactly hepatic fibrosis and cirrhosis have a causative role in HCC [2]. Indeed, the main strategy of HCC prevention is based on the elimination of the main causative etiologic factor: antihepatitis B and C virus vaccination, alcohol refusal, and lifestyle modification. However, often, secondary prevention strategy is required and antifibrotic therapy could be that one among all. Therapeutics targeting the signaling pathways involved in fibrogenesis (apoptosis signal-regulating kinase 1, peroxisome proliferator-activated receptors, and chemokine receptor inhibitors) are undergoing clinical trials and may serve as HCC chemoprevention [51]. So the obtained results allow us to propose the possible HCC-preventive activity of C_60_FAS.

The main mechanism of fibrous and malignant degeneration of liver tissue is ROS overproduction and antioxidant defense system depression, i.e., oxidative stress development [52]. ROS activate HSCs [4], whereby these cells adopt a myofibroblast-like phenotype. Activated HSCs proliferate and are characterized by high production of alpha smooth muscle actin, secretion of collagen types I and III, and expression of matrix metalloproteinases and their specific tissue inhibitors. Moreover, these cells play a role in inflammatory and immune-mediated responses, which can enhance hepatocellular necrosis and apoptosis and perpetuate the stimuli of fibrogenesis. Thus, activated HSCs secrete proinflammatory cytokines promoting the recruitment of inflammatory cells, express the adhesion molecules, and present antigens to T-lymphocytes and natural killer cells. Chemokines also promote the migration of activated HSCs to the site of injury, thereby boosting the inflammatory response [53] and oxidative stress perpetuation. In our studies, we observed the signs of oxidative stress in the liver manifested by elevated levels of MDA and PCG and decreased GP activity. Presumably, the increased ROS inhibits GP activity leading therefore to further escalation of the process [54]. However, we also observed the growth of GST activity. The increased expression of this enzyme is observed, in particular, in pulmonary fibrosis, which may be related to its involvement into the S-glutathionylation of FAS—a tumor necrosis factor (TNF) superfamily member, whose activation by binding the FAS ligand triggers apoptosis of the airway epitheliocytes. The latter leads to an imbalance between populations of epitheliocytes and myofibroblasts and stimulates fibrous tissue remodeling [55]. That is, an increase in GST activity may be a sign of myofibroblast activation and fibrous liver tissue degeneration. With the progression of the pathology, signs of oxidative stress persisted, however, changes in GP and GST activities were not so striking (tendency, *p* > 0.1). We did not observe a significant increase in GST activity while maintaining progressive liver tissue fibrotic degeneration probably due to exhausting in GSH pool—the substrate of the enzyme.

Since water-soluble nanoscale pristine C_60_ fullerenes are powerful antioxidants [56, 57], presumably, their ability to scavenge free radicals is one of the main mechanisms for providing antifibrotic activity. Indeed, the main three pathways responsible for liver fibrogenesis are growth arrest-specific gene 6 serum protein (Gas6)/Axl, transforming growth factor *β* (TGF-*β*)/Smad, and Wnt signaling [4], and all of them have been shown to be upregulated by ROS. Axl is a receptor tyrosine kinase expressed in several types of cells and is responsible for cell survival, migration, and growth. Gas6 is its endogenous ligand, binding of which promotes Axl tyrosine phosphorylation and activation. However, Axl may be activated not only by Gas6 but also by H_2_O_2_ (nonradical ROS) [58]. Moreover, Gas6 also increased production of ROS [59]. TGF-*β* is the most potent profibrogenic cytokine, and its expression is increased in almost all of fibrotic diseases. TGF-*β*1 increases ROS production in mitochondria and suppresses antioxidant enzymes, leading to a redox imbalance. ROS, in turn, induce/activate TGF-*β*1 and mediate many of TGF-*β*'s fibrogenic effects [60]. Wnt signaling is a well-established inflammatory and survival pathway. The canonical Wnt signaling pathway is driven by *β*-catenin, a scaffold protein, linking the cytoplasmic tail of classical cadherins. Without Wnt stimulation, cytoplasmic *β*-catenin levels are kept low by degradation. Binding of Wnt to its receptors leads to inhibition of *β*-catenin degradation enabling its accumulation and translocation to the nucleus for binding to several transcription factors. It was shown that low doses of H_2_O_2_ induce rapid stabilization of *β*-catenin and a concomitant increase in the expression of endogenous Wnt target genes [61, 62]. Furthermore, some studies evidenced that natural flavonoid and powerful antioxidant resveratrol alleviates fibrosis [4]. Therefore, we could suggest that C_60_ fullerene scavenging ROS might downregulate these pathways and thus inhibit fibrogenesis. Indeed, we demonstrated the decrease of the extent of lipid and protein peroxidation and restoration activity of antioxidant defense enzyme GP under C_60_FAS action suggesting mitigation of oxidative stress. Similar changes of GP activity under the action of C_60_FAS have been demonstrated in [63] and corroborated with our previous results [16]. We can explain the upregulation of this enzyme with the ability of C_60_ fullerene to scavenge ROS, creating therefore its optimal concentration for activating the enzyme. GST activity downregulation (compared to nontreated animals) under C_60_FAS administration may be due to a decrease in the number of activated myofibroblasts expressing it. Depressing the SOD and CAT activities in C_60_FAS-treated animals in parallel with obvious attenuation of oxidative stress (as evidenced by levels of MDA and PCG) might be caused by the ability of C_60_ fullerene to scavenge superoxide anion directly [64], thereby exhausting substrate pool for SOD and consequently CAT.

Although inflammation typically precedes fibrosis, the mechanisms that regulate fibrogenesis are distinct from those that regulate inflammation. Inflammation is characterized by a large infiltrate of mononuclear cells including macrophages, lymphocytes, eosinophils, and plasma cells. Lymphocytes are mobilized and stimulated by contact with antigen to produce lymphokines that activate macrophages. Cytokines from activated macrophages, in turn, stimulate lymphocytes, thereby setting the stage for persistence of the inflammatory response. There are two types of T-helper (Th) lymphocyte activation which results in equally potent inflammatory response but quite different effect on tissue fibrosis. Th1 lymphocytes produce interferon-*γ* (IFN-*γ*) and tumor necrosis factor alpha (TNF-*α*) and ultimately stimulate proinflammatory (M1) macrophages producing high levels of proinflammatory cytokines, such as TNF-*α*, interleukin-6 (IL-6), IL-12, IL-23, and matrix metalloproteases (MMP). Th2 lymphocytes produce IL-4 and IL-13, which stimulate anti-inflammatory (M2) macrophages promoting the resolution of inflammation, driving collagen deposition, coordinating tissue integrity, and releasing anti-inflammatory mediators [65, 66]. Actually, the properties of M1/M2 macrophages are similar to those of Th1/Th2 cells. However, liver macrophages cannot be sufficiently described as M1 or M2 cells. Thus, liver macrophages consist of two distinct populations: liver-resident phagocytes, or Kupffer cells (KCs), and bone marrow-derived recruited monocytes. KCs promote an immediate inflammatory response on injury (like M1) and attract monocyte-derived macrophages. The latter are derived from recruited lymphocyte antigen 6 complex, locus C (Ly6C)+monocytes, and have a promoting role in fibrogenesis producing profibrogenic factors, such as TGF-*β* and PDGF, which contribute to activation of HSCs (like M2). However, these cells exhibit a phenotypic switch to a tissue-restorative phenotype (Ly6C-) and are actively involved in fibrinolysis, primarily via the expression of MMP (like M1), which are critical for fibrotic matrix remodeling and degradation [67, 68]. As demonstrated in [69], water-soluble C_60_ fullerene could significantly increase the production of IL-12 and IFN-*γ* and suppress the production of IL-4 and IL-5 under induced immune response shifting immune response from Th2 to Th1. So we might conclude that something like this may occur in case of liver fibrosis: C_60_FAS induces shift of the immune response from Th2 to Th1 which leads to activation of M1-like (Ly6C-like) macrophage subpopulation followed by the increase of MMP production, fibrous tissue degradation, and eventually fibrosis resolution.

EGFR, VEGF, FGF, and PDGF signaling plays a pivotal role in HSC activation, proliferation, and liver fibrogenesis [6]. Overexpression of EGFR and FGFR by activated HSC and attenuation of hepatic fibrosis by depression of those have also been demonstrated [4, 70]. Using computational modeling, we showed the potential ability of C_60_ fullerene to bind with ATP-binding sites of EGFR and FGFR and to block the hinge displacement to avoid interaction of those with ATP. Hence, we suggest that C_60_ fullerene can inhibit liver fibrogenesis *in vivo* through EGFR and FGFR blockade and depressing their signaling. Moreover, in our previous study, we demonstrated the ability of C_60_ fullerene to inhibit EGFR expression in HepG2 cells [15].

Fibrotic tissue remodeling occurs after its damage and destruction of hepatocytes. We could assume that C_60_FAS slows down the processes of liver parenchyma injury and/or its replacement by connective tissue, although it does not completely avoid this process.

Although several antifibrotic drug candidates have recently been evaluated, no one is currently approved as specific antifibrotic treatment. Therapeutic interventions ongoing clinical trials include efforts to minimize hepatic injury and inflammation (corticosteroids, ursodeoxycholic acid, and hepatocytes' apoptosis inhibitors), to inhibit liver fibrogenesis by enhancing or inhibiting target receptor-ligand interactions or intracellular signaling pathways (interferons, antioxidants, angiotensin-converting enzyme inhibitors, TGF-*β* antagonists, and multitargeted kinase inhibitors), and to promote resolution of fibrosis (HSC-specific targeting) [71]. As anti-inflammatory [16], hepatoprotective [14, 15], and antifibrotic [17] effects of water-soluble pristine C_60_ fullerene have been demonstrated, this nanoparticle could be considered as a promising antifibrotic agent for treating liver diseases associated with its fibrosis.

## 5. Conclusions

We have shown that water-soluble pristine C_60_ fullerene treatment apparently reduces the connective tissue deposition in the liver and attenuates the degree of hepatocyte injury on simulated liver fibrosis and cirrhosis, hence inhibiting fibrogenesis. C_60_ fullerenes also mitigate oxidative stress manifestation in liver tissue and restore its redox balance. Moreover, they could bind to EGFR and FGFR and block them. This ability in addition to their antioxidant properties may contribute to C_60_ fullerene's antifibrotic action. Thus, the water-soluble nanoscale pristine C_60_ fullerenes may have a substantial therapeutic potential as an inhibitor of liver fibrosis and cirrhosis.

## Figures and Tables

**Table 1:** Fibrotic and other pathological alterations of the liver for rats that experienced DEN+CCl_4_ fibrosis and cirrhosis and treated by C_60_FAS.

Parameters	10 weeks			15 weeks		
	Control	Fibrosis	Fibrosis+C_60_FAS	Control	Cirrhosis	Cirrhosis+C_60_FAS
Liver damage score	0	8.2 ± 0.44^∗^	7.25 ± 0.5^∗#^	0	9.2 ± 0.8^∗^	7.8 ± 0.4^∗^
Ishak score	0	3.0 ± 0.88^∗^	0.75 ± 0.5^#^	0	5.4 ± 0.48^∗^	2.4 ± 1.2^∗#^
Lipid dystrophy of hepatocytes	-	+	+	-	++	++
Balloon dystrophy of hepatocytes	-	-	-	-	++	+
Ground-glass hepatocytes	-	+	-	-	++	-
Eosinophilic hepatocyte alteration	-	++	-	-	+	-
Hepatocellular hypertrophy	-	+	+	-	+	+
Necrotic hepatocytes	-	+	++	-	++	++
Blood vessel dilation	-	-	-	-	+	-
Inflammatory cell accumulation	+	+	+	+	++	++

Trait intensity: “-”: not observed; “+”: single or slight; “++”: moderate; “+++”: strong. ^∗^*p* < 0.05 compared to control; ^#^*p* < 0.05 compared to the fibrosis/cirrhosis group.

**Table 2:** Liver and plasma blood biochemical parameters for rats that experienced DEN+CCl_4_ fibrosis and cirrhosis and treated by C_60_FAS.

Parameters	10 weeks			15 weeks		
	Control	Fibrosis	Fibrosis+C_60_FAS	Control	Cirrhosis	Cirrhosis+C_60_FAS
Bilirubin conjugated (*μ*mol/l)	8.18 ± 1.63	7.94 ± 1.12	7.2 ± 1.11	14.49 ± 1.72	33.79 ± 3.84^∗^	16.54 ± 3.5^#^
Bilirubin nonconjugated (*μ*mol/l)	12.82 ± 3.7	8.26 ± 2.32	10.23 ± 1.41	21.15 ± 3.25	36.87 ± 1.63	11.22 ± 1.29^∗#^
ALT (*μ*mol/ml per h)	0.93 ± 0.06	0.8 ± 0.1	3.8 ± 0.46^∗#^	1.4 ± 0.17	4.1 ± 0.82^∗^	4.41 ± 0.3^∗^
AST (*μ*mol/ml per h)	0.81 ± 0.07	0.84 ± 0.12	2.99 ± 0.53^∗#^	1.45 ± 0.15	4.16 ± 0.45^∗^	3.66 ± 0.24^∗^
ALP (*μ*mol/ml per h)	23.14 ± 1.5	36.48 ± 2.0^∗^	29.42 ± 3.93	15.49 ± 2.54	69.07 ± 3.58^∗^	57.89 ± 8.02^∗^
LDH (U/l)	17.6 ± 2.11	57.04 ± 11.23^∗^	26.29 ± 8.02^#^	37.7 ± 8.7	1076.2 ± 201.6^∗^	416.1 ± 92.3^∗#^
Urea (mmol/l)	5.78 ± 0.27	7.31 ± 0.39^∗^	8.15 ± 0.47^∗^	4.22 ± 0.63	3.41 ± 1.06	7.9 ± 0.8^∗#^
Creatinine (*μ*mol/l)	95.0 ± 1.7	89.3 ± 8.9	128.4 ± 3.2^∗#^	98.8 ± 1.8	99.8 ± 4.5	123.3 ± 4.5^∗#^
*α*-Amylase (g/l per h)	168.1 ± 4.9	165.7 ± 5.5	159.7 ± 1.8	174.1 ± 3.6	163.9 ± 6.0	159.4 ± 6.8
SOD (U/mg protein)	64.64 ± 7.9	67.44 ± 17.0	36.58 ± 14.5	56.51 ± 11.39	70.71 ± 27.28	29.94 ± 7.27^∗#^
CAT (mol/mg protein)	0.83 ± 0.2	0.96 ± 0.15	0.48 ± 0.07^∗#^	0.46 ± 0.14	0.44 ± 0.16	0.51 ± 0.03
GSH (nmol/mg protein)	0.15 ± 0.02	0.13 ± 0.03	0.27 ± 0.1	0.27 ± 0.06	0.09 ± 0.01^∗^	0.07 ± 0.01^∗^
GST (*μ*mol/mg protein)	46.3 ± 8.2	111.0 ± 8.2^∗^	40.0 ± 7.2^#^	31.38 ± 6.59	53.32 ± 14.19	46.67 ± 8.75
GP (nmol/mg protein)	1.51 ± 0.3	0.54 ± 0.07^∗^	1.29 ± 0.29^#^	1.4 ± 0.36	1.09 ± 0.71	1.37 ± 0.21
MDA (*μ*mol/mg protein)	0.3 ± 0.04	0.4 ± 0.07	0.41 ± 0.07	0.35 ± 0.05	0.76 ± 0.26^∗^	0.42 ± 0.13
PCG (*μ*mol/mg protein)	45.9 ± 8.6	174.7 ± 48.9^∗^	44.2 ± 10.7^#^	42.41 ± 11.16	48.69 ± 11.14	33.75 ± 9.97

^∗^ *p* < 0.05 compared to control; ^#^*p* < 0.05 compared to the fibrosis/cirrhosis group.

## Data Availability

The datasets analyzed during the current study (computational modeling) are available in the PDB Protein Data Bank repository, https://www.rcsb.org/, and the UniProtKB UniProt Knowledgebase, https://www.uniprot.org/. Other relevant data used to support the findings of this study are included within the article.

## Abbreviations

HCC: Hepatocellular carcinoma
ROS: Reactive oxygen species
HSC: Hepatic stellate cell
C_60_FAS: Pristine C_60_ fullerene aqueous colloid solution
AFM: Atomic force microscopy
DLS: Dynamic light scattering
DEN: N-Diethylnitrosamine
H&E: Hematoxylin and eosin
ALT: Alanine aminotransferase
AST: Aspartate aminotransferase
ALP: Alkaline phosphatase
LDH: Lactate dehydrogenase
PBS: Phosphate-buffered saline
EDTA: Ethylenediaminetetraacetic acid
PMSF: Phenylmethylsulphonyl fluoride
MDA: Malonic dialdehyde
PCG: Protein carbonyl groups
GSH: Reduced glutathione
SOD: Superoxide dismutase
CAT: Catalase
GP: Glutathione peroxidase
GST: Total glutathione-S-transferase
NBT: Nitro blue tetrazolium
EGF(R): Epidermal growth factor (receptor)
VEGF(R): Vasoendothelial growth factor (receptor)
FGF(R): Fibroblast growth factor (receptor)
PDGF(R): Platelet-derived growth factor (receptor)
PDB: Protein Data Bank
MD: Molecular dynamics
QXP: Quick explore
Gas6: Arrest-specific gene 6
TGF-*β*: Transforming growth factor *β*
IFN-*γ*: Interferon-*γ*
TNF-*α*: Tumor necrosis factor alpha
KC: Kupffer cell
MMP: Metalloproteases
Ly6C: Lymphocyte antigen 6 complex, locus C.
